# Supplementary material for: Deep learning-based diagnosis of feline hypertrophic cardiomyopathy
Source: PLoS One. 2023 Feb 2;18(2):e0280438. doi: 10.1371/journal.pone.0280438 (PMC9894403; doi:10.1371/journal.pone.0280438)
Supplement: S1 Table — (DOCX) [file pone.0280438.s001.docx]

**S1 Table**

|  | Train | Test | Peeking analysis | Total |
| --- | --- | --- | --- | --- |
| Normal | 88 | 11 | 10 | 109 |
| HCM | 143 | 11 | 10 | 164 |
| Sum | 231 | 22 | 20 | 273 |
