# Supplementary material for: Deep learning-based diagnosis of feline hypertrophic cardiomyopathy
Source: PLoS One. 2023 Feb 2;18(2):e0280438. doi: 10.1371/journal.pone.0280438 (PMC9894403; doi:10.1371/journal.pone.0280438)
Supplement: S2 Table — (DOCX) [file pone.0280438.s002.docx]

**S****2 Table**

|  | ResNet50V2 | Resnet152 | InceptionResnetV2 | MobilenetV2 | Xception |
| --- | --- | --- | --- | --- | --- |
| New data | 75 | 70 | 85 | 55 | 80 |
| Combined data | 86 | 83 | 90 | 76 | 86 |
