# Supplementary material for: Deep learning-based diagnosis of feline hypertrophic cardiomyopathy
Source: PLoS One. 2023 Feb 2;18(2):e0280438. doi: 10.1371/journal.pone.0280438 (PMC9894403; doi:10.1371/journal.pone.0280438)
Supplement: Attachment — Submitted filename: Response to Reviers.docx [file pone.0280438.s003.docx]

[2022.09.16.]

Emily Chenett

Editor-in-Chief

*PLOS ONE*

Dear Editor:

We wish to re-submit the manuscript titled ““Deep learning-based diagnosis of feline hypertrophic cardiomyopathy: Comparison of five neural engines.” The manuscript ID is D-22-14859.

We thank you and the reviewers for your thoughtful suggestions and insights. The manuscript has benefited from these insightful suggestions. I look forward to working with you and the reviewers to move this manuscript closer to publication in *PLOS ONE.*

The manuscript has been rechecked and the necessary changes have been made in accordance with the reviewers’ suggestions. The responses to all comments have been prepared and attached herewith/given below.

Thank you for your consideration. I look forward to hearing from you.

Sincerely,

Hwa-Young Son

College of Veterinary Medicine,

Chungnam National University, Daejeon,

Republic of Korea

E-mail: hyson@cnu.ac.kr

Reviewer #1: Summary: Rho et al attempt to use deep learning networks to diagnose hypertrophic cardiomyopathy from radiographs of cats. Networks were trained on 231 radiographs (143 HCM vs 88 normal) and tested on a hold-out dataset of 22 radiographs (11 HCM vs 11 normals). Five different off-the-shelf network types were compared and all five gave identical results: 95% diagnostic accuracy (compared to 75% without AI).

Review:
I disclose that I have no expertise in vetenary/feline medicine but there does seem to be significant utility in such an algorithm. The manuscript is easy to follow and understand.

There are however some limitations:
1. the numbers of both training and test data (only 11 diseased subjects) is small. Probably too small to judge the algorithm's effectiveness on.

Thank you for your advice. We understand your concerns regarding the small test datasets. Because of the scarcity of legally available radiographic images of HCM-affected cats, we had no choice but to focus on training the model. To enhance the credibility of our model, additional 10 radiographic images of normal and HCM-affected cats from another animal hospital are evaluated.

2. the validation set seems to be from the same institution and not independent, which limits the generalisability of the conclusions.

Thank you for the critical advice on the generalizability of the image. The image is obtained not only from the teaching hospital but also from three other local hospitals. We did not comment on the data's origin for the ethical and private concerns. Here, we included the tables describing the origin of the images (although the specific name of the institution is hidden).

| Animal hospital  Case | A | B | C | D | E  (revision) | Total |
| --- | --- | --- | --- | --- | --- | --- |
| Normal | 32 | 30 | 27 | 10 | 10 | 109 |
| HCM | 55 | 50 | 34 | 15 | 10 | 164 |

3. the paper only compares healthy cats to HCM. In humans, this is an easy problem -- the difficulty comes when you also consider other pathologies/phenotypes such as DCM, valve disease etc which probably give a similar cardiac silhouette. I would be interested to know how the algorithm performs on other pathologies.

We appreciate your advice. The prevalence of cardiac disease in cat is quite different from any other animals or human. The most frequent, predominant cardiac disease is HCM and other cardiac disease is very rare in cat. In 306 primary cardiac disorders from 1998 to 2005, 252 cases were cardiomyopathy (82%) and 48 cases (16%) were congenital heart disease. We unfortunately failed to obtain radiographic images of other cardiac diseases in cats. Therefore, diagnosing whether it is DCM from radiographic image may be most important for cats.

S.C. Riesen, A. Kovacevic, et al. Prevalence of heart disease in symptomatic cats: an overview from 1998 to 2005

Richard W. Nelson, C. Guillermo Couto, Small animal internal medicine 5^th^ edition.

4. they use the opinion of an experienced reader as ground truth -- this is not very strong. Wouldn't echo/ultrasound give you a much stronger gold standard?

We thank you for the interesting question. As mentioned, the gold standard for diagnosing heart disease is an ultrasound.

Because only specialized veterinarians can perform echocardiography and a US machine is required, most veterinarians rely on X-ray and auscultation. The animal is referred to a specialized animal hospital if cardiac disease is suspected in auscultation or radiography. At this point, the deep learning algorithm we developed can suggest and screen whether the animal may be affected by HCM at the x-ray level.

5. the comparison of 5 network architecture does not add much -- particularly since results are identical.
Thank you for the advice. As the accuracy of the architecture is high predominantly, comparing the five models seems futile. However, in the detailed observation, you will see that misdiagnosed images are different except Resnets. The data shows that each model has different flaws that drive the misdiagnosis.

Based on your opinion that the results are identical, we ensembled the trained architecture. The ensemble model works when the prediction made by each model considers five prediction values and makes the final decision. As a result, the ensemble model achieved 100% accuracy from the old test sample.

Minor comments
1. while the paper is easy to follow there are several grammatical errors which could do with correction

Thank you for the detailed advice. We requested a re-assessment of the English and modified it accordingly.

2. were images augmented for training? Presumably so given the small size of the training data but the parameters need to be stated.

Thank you for the specific advice. We strengthened the detailed description of data augmentation.

3. Several performance measures are reported (e.g. in table 1), but sensitivity and specificity would be most useful

Thank you for the thorough suggestion. We added sensitivity and specificity to the table1.

4. please include more details on ethics.

We appreciate your advice. We elaborated on the details of the ethics.

Reviewer #2: As its name indicates, this paper deals with Deep learning-based diagnosis of feline hypertrophic cardiomyopathy through Comparison of five neural engines

1. I do not have any comments about the deep models, but the outcomes of the simulations are given only quantitatively. It would be nice to have some expert feedbacks for evaluating the results.

2. The authors should also show that the results explained in this article have fair developing stages without making “**peeking**”. Briefly, peeking is using testing datasets for validation purposes (such as parameter tuning) by making too many iterative submissions (Kuncheva (2014), page 17). In other words, testing sets, which should only be in the previously unseen data, now do not serve testing purposes anymore. Even though it is an indirect usage, peeking is surprisingly an underestimated problem in academic studies which causes overfitting of deep models on a target data. Therefore, theoretically very successful models for specific data may not be useful for real-world problems.

Kavur, A. Emre, et al. "CHAOS challenge-combined (CT-MR) healthy abdominal organ segmentation." Medical Image Analysis 69 (2021): 101950.

**The authors should at least discuss the potential effects of peeking on their results.

They should also consider, (maybe apply if possible) and discuss strategies that are being used to avoid peeking such as:**

Selver. et al. "Basic Ensembles of Vanilla-Style Deep Learning Models Improve Liver Segmentation From CT Images." arXiv preprint arXiv:2001.09647 (2020).

Conze, Pierre-Henri, et al. "Abdominal multi-organ segmentation with cascaded convolutional and adversarial deep networks." Artificial Intelligence in Medicine 117 (2021): 102109.

Thank you for the crucial advice. As mentioned, peeking is a crucial obstacle when we develop the model. To avoid peeking, we divided the validation sample from the training sample during the training session and tested it on untrained samples. In addition, we obtained additional radiographic samples from another vet hospital and tested them. Although the sample accuracy obtained was not very precise, we achieved over 78% accuracy from additional samples.

We added these findings to the results section.
3. Since the authors used many models to compare the results, they can also test the **ensemble** strategies, which are shown to outperform single model results at medical image analysis applications such as :
We appreciate your important advice. As you mentioned, the ensemble strategy can outperform a single model when every single neural model shows high variance in its prediction. We applied a voting ensemble to five models and achieved 100% accuracy from the test sample. The additional sample showed only 80% accuracy, although the single model (Xception) achieved 97% accuracy.

This finding has been added to the results section.

Menze, Bjoern H., et al. "The multimodal brain tumor image segmentation benchmark (BRATS)." IEEE transactions on medical imaging 34.10 (2014): 1993-2024.

Kavur, A. Emre, et al. "Comparison of semi-automatic and deep learning-based automatic methods for liver segmentation in living liver transplant donors." Diagnostic and Interventional Radiology 26.1 (2020): 11.

4. The diversity and omplementarity of the utilized models shoudl also be analyzed through statistis and Kappa. An eemplary analysis an be found at

Toprak, Tugce, et al. "Conditional weighted ensemble of transferred models for camera based onboard pedestrian detection in railway driver support systems." IEEE Transactions on Vehicular Technology 69.5 (2020): 5041-5054.

Thank you for the critical advice for our research. In our opinion, the kappa analysis may be helpful in cases wherein there is no exact answer to the problem (ex, unsupervised training). However, the analysis is not applicable in this study because vet specialists confirm all the data. We already compared the results of each model and provided the accuracy of each model.
