# Supplementary material for: Deep learning-based diagnosis of feline hypertrophic cardiomyopathy
Source: PLoS One. 2023 Feb 2;18(2):e0280438. doi: 10.1371/journal.pone.0280438 (PMC9894403; doi:10.1371/journal.pone.0280438)
Supplement: Attachment — Submitted filename: Response to Reviers.docx [file pone.0280438.s004.docx]

[2022.12.09.]

Dear Editor

*PLOS ONE*

Dear Editor:

We wish to re-submit the manuscript titled ““Deep learning-based diagnosis of feline hypertrophic cardiomyopathy: Comparison of five neural engines.” The manuscript ID is D-22-14859.

We thank you and the reviewer for your considerate suggestions and advice. The manuscript has benefited from these insightful suggestions. I look forward to working with you and the reviewers to move this manuscript closer to publication in *PLOS ONE.*

The manuscript has been rechecked and the necessary changes have been made in accordance with the reviewers’ suggestions. The responses to all comments have been prepared and attached herewith/given below.

Thank you for your consideration. I look forward to hearing from you.

Sincerely,

Hwa-Young Son

College of Veterinary Medicine,

Chungnam National University, Daejeon,

Republic of Korea

E-mail: hyson@cnu.ac.kr

Reviewer #1: Thank you to the authors for taking our comments on board and adapting the manuscript -- a lot of effort has gone into this the manuscript is improved as a result.

In summary, the test data has expanded, but still on the small side (though I appreciate the scarcity of data) and the ground truth is slightly suboptimal (but consistent with what is used in clinical practice). The authors have answered all my comments and I have responded to these, with my responses denoted by '>>'.

1.. the numbers of both training and test data (only 11 diseased subjects) is small.
Probably too small to judge the algorithm's effectiveness on.
- Thank you for your advice. We understand your concerns regarding the small test
datasets. Because of the scarcity of legally available radiographic images of HCMaffected cats, we had no choice but to focus on training the model. To enhance the
credibility of our model, additional 10 radiographic images of normal and HCM-affected
cats from another animal hospital are evaluated.
>> thank you. It would be worth emphasising in the main text that you now have more independent test data (the text still reads 11 HCM and 11 normals).

- Thank you for the advice. We changed the main text to 42 images used in the test dataset.

2. the validation set seems to be from the same institution and not independent, which
limits the generalisability of the conclusions.
- Thank you for the critical advice on the generalizability of the image. The image is
obtained not only from the teaching hospital but also from three other local hospitals.
We did not comment on the data's origin for the ethical and private concerns. Here, we
included the tables describing the origin of the images (although the specific name of
the institution is hidden) in supplementary table 1.
>>thanks. it would be well worth emphasising this in the main text.

- thank you. The generalizability of the data is added to the abstract and the materials and methods. To emphasize the five institutions, we moved supplementary table 1 into table 1.

3. the paper only compares healthy cats to HCM. In humans, this is an easy problem --
the difficulty comes when you also consider other pathologies/phenotypes such as
DCM, valve disease etc which probably give a similar cardiac silhouette. I would be
interested to know how the algorithm performs on other pathologies.
- We appreciate your advice. The prevalence of cardiac disease in cat is quite different
from any other animals or human. The most frequent, predominant cardiac disease is
HCM and other cardiac disease is very rare in cat. In 306 primary cardiac disorders
from 1998 to 2005, 252 cases were cardiomyopathy (82%) and 48 cases (16%) were
congenital heart disease. We unfortunately failed to obtain radiographic images of
other cardiac diseases in cats. Therefore, diagnosing whether it is HCM from
radiographic image may be most important for cats.
S.C. Riesen, A. Kovacevic, et al. Prevalence of heart disease in symptomatic cats: an
overview from 1998 to 2005
Richard W. Nelson, C. Guillermo Couto, Small animal internal medicine 5th edition.
>>thanks for clarifying -- this makes sense. Could this information (and the references) be added to the main body of the paper?

- thank you for the advice. this information is added to the introduction.

4. they use the opinion of an experienced reader as ground truth -- this is not very
strong. Wouldn't echo/ultrasound give you a much stronger gold standard?
- We thank you for the interesting question. As mentioned, the gold standard for
diagnosing heart disease is an ultrasound.
Because only specialized veterinarians can perform echocardiography and a US
machine is required, most veterinarians rely on X-ray and auscultation. The animal is
referred to a specialized animal hospital if cardiac disease is suspected in auscultation
or radiography. At this point, the deep learning algorithm we developed can suggest
and screen whether the animal may be affected by HCM at the x-ray level.
>>OK, thanks. Like me, many PLOS readers will also have little expertise in feline medicine and the availability of data so it would be worth emphasising this in the text. It would also be worth adding a discussion about the limitations (subjectivity, error and variability of experts etc) in the discussion.

- thank you for the advice. The feline medicine and the availability of the data is added into the discussion.

5. the comparison of 5 network architecture does not add much -- particularly since
results are identical.
- Thank you for the advice. As the accuracy of the architecture is high predominantly,
comparing the five models seems futile. However, in the detailed observation, you will
see that misdiagnosed images are different except Resnets. The data shows that each
the model has different flaws that drive the misdiagnosis.
Based on your opinion that the results are identical, we ensembled the trained
architecture. The ensemble model works when the prediction made by each model
considers five prediction values and makes the final decision. As a result, the
ensemble model achieved 100% accuracy from the old test sample.
>>Thanks. You might want to reconsider the title etc. The main contribution to the paper is a novel algorithm for diagnosing feline HCM from X-rays. Adding the subtitle about 5 neural nets downplays your contribution a little...

- Thank you for the advice. We changed the title so that the novel algorithm and the ensemble method are focused.
